# Extraction and purification of antioxidative flavonoids from *Chionanthus retusa* leaf

**DOI:** 10.3389/fbioe.2022.1085562

**Published:** 2022-12-09

**Authors:** Zhen Wang, Shilong Yang, Yajun Gao, Jianting Huang

**Affiliations:** ^1^ Lianyungang Forestry Technical Guidance Station, Lianyungang, China; ^2^ Advanced Analysis and Testing Center, Nanjing Forestry University, Nanjing, China

**Keywords:** *Chionanthus retusa* leaves, flavonoids, extraction, purification, antioxidant activity

## Abstract

In this work, flavonoids from the leaves of *Chionanthus retusa* were extracted using alcohol, and the extraction yield was optimized by single-factor and orthogonal experiments. Then, the extracted solution with flavonoids was purified *via* macroporous resin by elution with different concentrations of ethanol. The antioxidative activity of total flavonoid in purified extracted solution was evaluated by detecting its ability to scavenge DPPH free radicals. The results demonstrated that ethanol with a concentration of 60%, ultrasonic power of 140 W, liquid–solid ratio of 25:1 ml g^−1^, and water-bath temperature of 80°C were the optimal conditions for the extraction of total flavonoids from *C. retusa* leaf, achieving a yield of 121.28 mg g^−1^. After purification by macroporous resin using different concentrations of ethanol, the highest content of total flavonoids (88.51%) in the extracted solution can be obtained with the 50% ethanol eluant. The results of scavenging DPPH free radicals suggest that the purified flavonoids in the 50% ethanol eluant had the best antioxidant capacity over the flavonoids in other ethanol eluants. In addition, it is confirmed the antioxidant capacity of the extractives was associated with the content of total flavonoids and kinds of flavonoids. These results may provide a feasible pathway to make full use of total flavonoids from *C. retusa* leaf.

## Introduction

*Chionanthus retusus Lindl. et Paxt.* (*Chionanthus retusus*) is a woody biomass that is distributed throughout East Asia and North America, including China, South Korea, Japan, North Korea, and the United States. Nowadays, most *C. retusus* trees are planted for rootstock and street trees, and produce a large scale of potential leaf resources. The leaf of *C. retusus* has been used for hundreds of years in China to treat sunstroke, diarrhea, stomachache, and indigestion (Arias et al., 2011; Chen et al., 2018; Pei et al., 2020). However, the leaves of street trees are regarded as waste biomass and are just burned or put into landfill. However, these leaves are very useful when their effective extractives are considered.

Current studies show that many kinds of active ingredients, including flavonoids, polyphenols, functional polysaccharides, lignans, and terpene compounds, have been extracted from *C. retusus* trees (Gulcin et al., 2006; Gulcin et al., 2009; Seo et al., 2015; Gao and Yin, 2016; Kwak et al., 2019; Wang et al., 2021). Many reports have demonstrated that flavonoids have many beneficial biological properties, such as anti-oxidation, anti-inflammatory, anti-viral, and antitumor properties (Liu et al., 2015; Lee Y. et al., 2019; Lee Y. G. et al., 2019). It has been shown that many flavonoids exist in *C. retusus* leaf (Hu et al., 2012). In addition, the flavonoids from its flower could efficiently scavenge the free radicals of O_2_^−^, OH, and DPPH and reactive oxygen species (Sun et al., 2015; Pei et al., 2020), which are related to various diseases in human body (Deng et al., 2022; Liu et al., 2022; Pei et al., 2022). In the work of Lim et al. (2004) an extract of *C. retusa* leaf was made with various solvents to investigate antioxidative activity by scavenging DPPH radicals. This further characterization showed that luteolin-4′-O -glucoside was isolated from acetate fraction, demonstrating effective DPPH radical scavenging activity. Further studies have revealed that biological activities are highly dependent on the content of flavonoids in samples (Aryal et al., 2019). Hence, it is worth isolating the antioxidant extract from *C. retusa* leaf. The current literature has just focused on the investigation of the antioxidative ability of crude extractive from *C. retusa* leaf. However, due to the complex components in the extractive solution, it is necessary to isolate the related pure components in *C. retusa* leaf to understand its antioxidative ability.

Generally, crude flavonoids extracted from biomass contain many kinds of compounds—such as proteins, terpenoids, pigments, and carbohydrates—which could affect biological activities (Zhao et al., 2021). Thus, it is essential to improve the proportion of flavonoids in an extract. Purification methods include macroporous resin column chromatography, organic solvent extraction, gel chromatography, and co-precipitation. Among these, macroporous resin column chromatography technology is a popular method can be recycled and has good selectivity, low cost, and nontoxicity (Shen et al., 2022). In addition, macroporous resin column chromatography is suitable for the large-scale preparation of flavonoids by increasing the quantity of macroporous resin (Hu et al., 2022). Thus, macroporous resin column chromatography is an efficient method for the purification of flavonoids. However, there has been little work on obtaining pure flavonoids from the *C. retusa* leaf to investigate its antioxidative ability.

In this work, single-factor and orthogonal experiments were used extract flavonoids from *C. retusa* leaf by ultrasonic-assisted ethanol extraction. Meanwhile, macroporous resin column chromatography was employed to separate and purify flavonoids in extracted solution. Finally, the antioxidative activity of purified flavonoids was evaluated by analyzing their ability to scavenge DPPH free radicals. It is hoped that this work can provide an effective approach to extracting, separating, and purifying flavonoids from *C. retusa* leaf.

## Materials and methods

### Reagents

Analytical grade ethanol, vitamin C (VC), and sodium nitrite (NaNO_2_) were purchased from Sinopharm Chemical Reagent Co., Ltd. Rutin (>98%) and 1,1-diphenyl-2-picrylhydrazyl (DPPH) were obtained from Aladdin Reagent Co., Ltd. (Shanghai, China). Aluminum nitrate (Al(NO_3_)_3_) and sodium hydroxide (NaOH) were analytical grade and provided by the Nanjing Reagent Co., Ltd., *C. retusus* leaves were collected on the Yuntai Mountain in Lianyungang, Jiangsu province. Purified water was obtained from Milli-Q system (Millipore, America). Ethanol solution with different concentrations was prepared according to volume ratio. HPD5000 macroporous resin was obtained from Cangzhou Bon Adsorber Technology Co., Ltd.

### Extraction of total flavonoids

Dried powder (1 g) of *C. retusus* leaf was extracted by using ethanol solution in a flask with an ultrasonic bath for 30 min. The extract solution was separated from the powder by centrifugation, and the powder was continually extracted with ethanol solution twice. The extract solution was merged in a volumetric flask and diluted to 100 ml with 50% ethanol solution.

### Determination of total flavonoid content

The total flavonoid content (TFC) was determined by a modified NaNO_2_-Al(NO_3_)_3_-NaOH colorimetric method (Hou et al., 2020). A standard rutin solution of 0.1 mg mL^−1^ was prepared, and an appropriate volume of standard rutin solution was added into different 10-ml volumetric flasks. Subsequently, solutions of 5% NaNO_2_ (0.3 ml), 10% Al(NO_3_)_3_ (0.3 ml), and 4% NaOH (4 ml) were added at 5 min interval. Afterward, the mixed solutions were diluted to 10 ml with 50% ethanol. In the final solutions, the concentration of rutin was 0, 1, 5, 10, 15, 20, 25, 30, 35, 40, 45, and 50 μg mL^−1^. Absorbance of the solution was measured on Lambda 950 spectrophotometer at 510 nm thrice. The absorbance was in direct proportion to rutin concentration, ranging from 0 to 50 μg mL^−1^. The calibration curve is shown in Supplementary Figure S1; the equation was A = 0.0127C + 0.0022 (*R* ^*2*^ = 0.9994), where A was average absorbance of the respective solution and C was the concentration of rutin.

TFC in the extract solution was determined according to the above method by replacing rutin with the extract solution. Extraction yield was expressed as TFC in *C. retusus* leaf, which was calculated according to the calibration curve and expressed as mg rutin equivalent per gram weight of dried leaf (mg g^−1^).

### Single-factor experiments

The extraction conditions of the total flavonoids from *C. retusus* leaf were optimized by single-factor experimentation (Mitic et al., 2021). The impact factors including ultrasonic power, liquid–solid ratio, concentration of ethanol solution, and extraction temperature were investigated in this study.

Dried powder (1 g) of *C. retusus* leaf was extracted by ethanol solution in a flask with an ultrasonic bath for 30 min. When the effect of the ultrasonic power (80 W, 100 W, 120 W, 140 W, 160 W, 180, and 200 W) was evaluated, the concentration of ethanol solution was 50%, the extraction temperature was heated to 60°C, and the liquid–solid ratio was controlled at 20:1 ml g^−1^. When optimizing the concentration of the ethanol solution, the concentration of ethanol was 0%, 20%, 40%, 80%, and 100%. The temperature was 60 °C, the liquid–solid ratio was 20:1 ml g^−1^, and the ultrasonic power was 120 W. When studying the function of the liquid–solid ratio, the ratio was set at 5:1, 10:1, 15:1, 20:1, 25:1, 30:1 ml g^−1^. The concentration of ethanol solution was 60%, the extraction temperature was 60°C, and the ultrasonic power was 120W. When investigating the influence of temperature on the extraction progress, the temperature was chosen at 30°C, 40°C, 50°C, 60°C, 70°C, 80°C, and 90°C. The liquid–solid ratio was 20:1 ml g^−1^, the concentration of ethanol solution was 60%, and the ultrasonic power was 120 W. The extract solution was treated according to the steps in 2.2. The TFC was measured according to the steps in 2.3.

### Orthogonal experiments

To further optimize the parameters for extracting total flavonoid from *C. retusus* leaf, the extraction process was optimized by the orthogonal experiments through a single-factor experiment (Zhu et al., 2011). Orthogonal experiments were performed using a L_9_ (3^4^) orthogonal design to optimize the effects of ethanol concentration (A), extraction temperature (B), liquid-solid ratio (C), and ultrasonic power (D) on TFC in *C. retusus* leaf (Table 1).

**TABLE 1:** Design of orthogonal experiment.

Levels	Factors			
	Ethanol concentration (A)	Extraction temperature (B)	Liquid-solid ratio (C)	Ultrasonic power (D)
1	40	60	15:1	100
2	60	70	20:1	120
3	80	80	25:1	140

### Purification of total flavonoids

To improve TFC in *C. retusus* leaf extract, macroporous resin HPD500 was used to separate and purify the total flavonoids (Hou et al., 2019). The extract solution was evaporated by vacuum distillation to remove ethanol, and then plenty of water was added until the extract solution was almost clear. To ensure that total flavonoids were adsorbed by macroporous resin, the diluent extract solution was loaded onto a macroporous adsorbing resin column and trickled into a beaker with flow velocity of 1 ml min^−1^. A gradient concentration of ethanol–water solution (0%–60%) was used to elute with a flow velocity of 3 ml min^−1^ (Shen et al., 2019). The eluant was collected and evaporated to remove ethanol, and the solid-state extract was obtained by vacuum freeze-drying.

### DPPH radical scavenging assay

*In vitro* antioxidant activity was explored by DPPH radical scavenging assay according to the documentation with some modification (Chen et al., 2022). The extract solution was prepared by adding 20 mg dried extract obtained in 2.6 into a 10 ml ethanol–water solution (50%) and diluted into different concentrations. Dilute extract solution of 1 ml was transferred into a volumetric flask (10 ml) containing DPPH solution, sequentially diluted with ethanol to volume. After half an hour, the absorbance obtained at 517 nm was expressed as A_i_. When DPPH solution in a volumetric flask was replaced by the same volume of ethanol solution, the absorbance obtained at 517 nm was expressed as A_j_. When the sample solution in a volumetric flask was replaced by the same volume of ethanol solution, the absorbance obtained at 517 nm was expressed as A_0_. All absorbance was recorded thrice and calculated as a mean value. The inhibition rate (I%) of DPPH was calculated using the following formula:

$$I\%=\left[1-\left(A_{i}-A_{j}\right)/A_{0}\right]\times 100\%$$

## Results and Discussion

### Single-factor experiments

**FIGURE 1:**
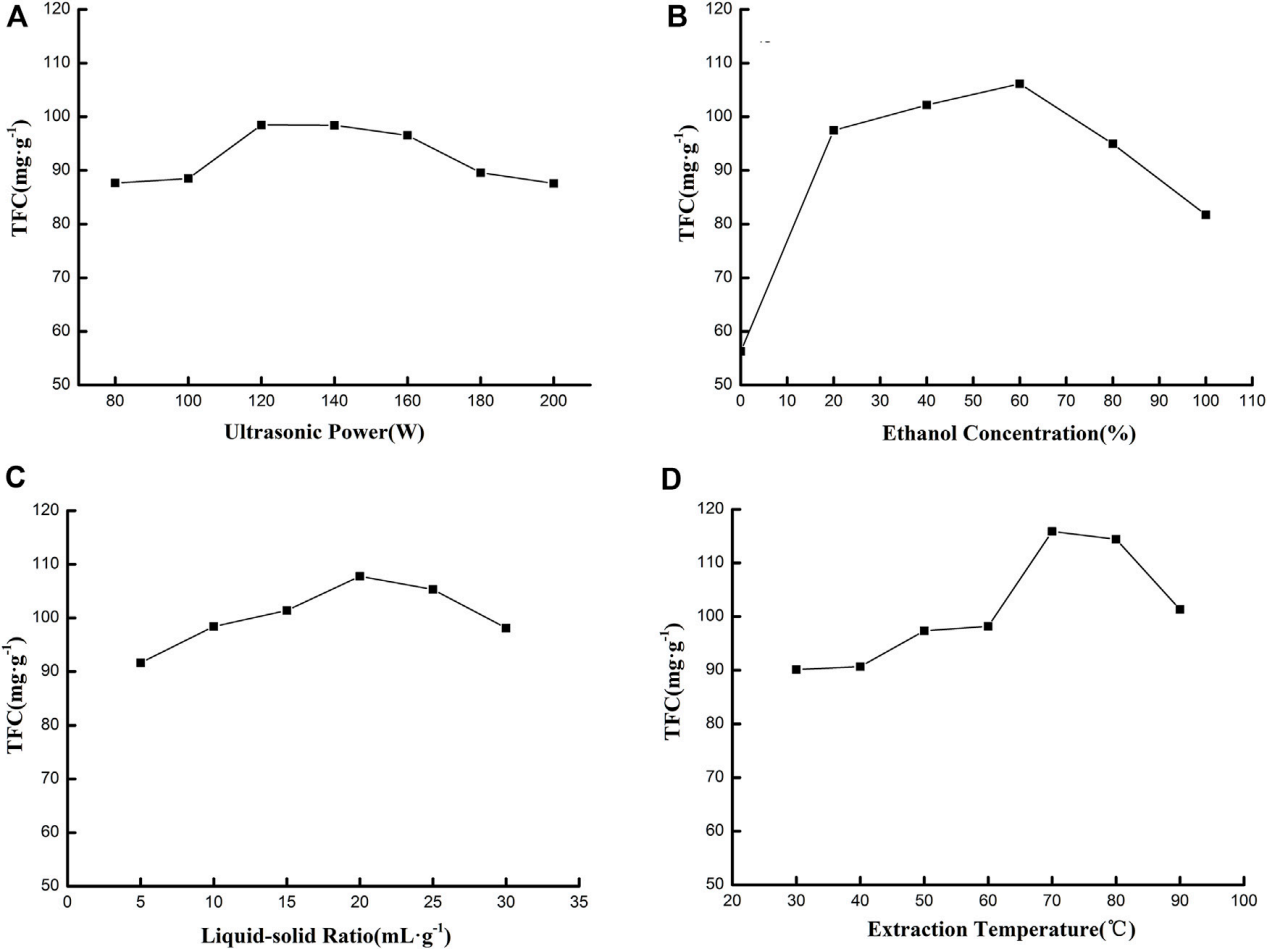
Effect of ultrasonic power **(A)**, ethanol concentration **(B)**, liquid-solid ratio **(C)**, and extraction temperature **(D)** on TFC.

The impact factors, which included ultrasonic power, liquid-solid ratio, concentration of ethanol solution, and extraction temperature, were optimized by a single-factor experiment. All results are exhibited in Figure 1. Figure 1A illustrates the effects of ultrasonic power on TFC. When the ultrasonic power range was 80–200 W, TFC increased and then decreased—the highest TFC was 98.46 mg g^−1^. Ultrasonic power affected the cavitation effect that would contribute to improving TFC in extracting solution. With increased ultrasonic power, the cavitation effect was continuously strengthened, resulting in cell wall rupture and faster exchange between the solvent and solute. Therefore, more flavonoids were extracted from the leaf. However, when ultrasonic power was too strong, large quantities of other chemical compounds (protein, polysaccharide, etc.) were also extracted; local high temperature could also result, which would destroy the structure of flavonoids. These problems could inhibit the extraction process of flavonoids, resulting in a decreased extraction yield (Liu et al., 2021). Thus, ultrasonic power was fixed 120 W for the extraction.

The effect of ethanol concentration on TFC is shown in Figure 1B. TFC became higher and then lower with an ethanol concentration range of 0%–100%. The highest amount that TFC rose to was 106.15 mg g^−1^ when the ethanol concentration was 60%. Flavonoid compounds generally existed as flavone glycoside and flavonoid aglycones, which possessed different polarities. Thus, the solvent's polarity was vital for the extraction process. Various concentrations of ethanol solution had a different polarity, which could extract flavonoids at a specific polarity. The polarity of ethanol solution was inversely proportional to ethanol concentration. When ethanol concentration was lower, the polarity of ethanol solution was strong, and only polar flavone glycoside would be extracted. When ethanol concentration was higher, the polarity of ethanol solution became weak, and only weak polar flavonoid aglycones could be extracted. It was when ethanol concentration was 60% that the polarity was suitable. Both flavone glycoside and flavonoid aglycones could be maximally extracted , resulting in the highest TFC (Lim et al., 2019). Consequently, the 60% ethanol concentration was chosen to extract total flavonoids.

Figure 1C shows the impact of the liquid–solid ratio on the TFC. It was found that this ratio affected TFC significantly. An increasing trend of TFC was observed with a liquid–solid ratio increasing from 5:1 to 20:1 ml g^−1^, and a decreasing trend appeared with the ratio increasing from 20:1 to 30:1 ml g^−1^, giving a maximum TFC of 107.76 mg g^−1^. Generally, the TFC increased with an increasing liquid–solid ratio due to high mass transfer between the leaf and the ethanol solution at a higher liquid–solid ratio. Meanwhile, the solubility of flavonoids also limited TFC; more flavonoids could be extracted more easily with more ethanol solution. However, at a further increase of the liquid–solid ratio, TFC declined gradually. Therefore, 20:1 ml g^−1^ was chosen for the remaining experiments.

The impact of extraction temperatures of 30°C–90°C was studied. As shown in Figure 1D, it was demonstrated that TFC increased with extraction temperatures of 30°C–70°C. However, when this temperature rose from 70°C to 90°C, TFC decreased gradually. The illustration indicates that the highest TFC was 114.43 mg g^−1^ at 70°C. At the beginning of heating, there was a high mass transfer between the leaf and the ethanol solution due to molecular motion, which increased the diffusivity and solubility of flavonoid compounds in the ethanol solution. At high temperature, flavonoid compounds might be oxidized and easily degrade. In addition, the extraction yield of other compounds also increased quickly, which inhibited the extraction of flavonoids and led to lower TFC (Wang et al., 2017). Thus, the optimal temperature was 70°C for extraction.

The results of the single-factor experiment demonstrated that the optimal conditions for extracting flavonoids from *C. retusus* leaf were as follows: the dried powder of the leaf was extracted by 60% ethanol solution in a 120 W ultrasonic bath for 30 min at an extraction temperature of 70°C and liquid–solid ratio of 20:1 ml g^−1^.

### Orthogonal experiment

To further optimize the conditions of ethanol concentration (A), extraction temperature (B), liquid-solid ratio (C), and ultrasonic power (D), a four-factor and three-level (L_9_ (3^4^)) orthogonal experiment was designed on the basis of the results obtained from the single-factor experiment. The range-analysis results are shown in Table 2. It was evident that the factors were important in the following descending order: A > D > C > B. That is, ethanol concentration was the most important factor for extraction process, followed by ultrasonic power, then liquid–solid ratio, and finally extraction temperature. Based on the orthogonal experiment results, the optimal conditions were A_2_D_3_C_3_B_3_, meaning that the optimal conditions for extracting total flavonoids from *C. retusus* leaves are follows: ethanol concentration of 60%, ultrasonic power of 140 W, liquid–solid ratio of 25:1 ml g^−1^, and extraction temperature of 80°C. To evaluate the accuracy of the optimal conditions, three parallel experiments for extracting total flavonoids from *C. retusus* leaf were conducted under the optimal conditions; the results in Table 3 show TFC reaching up to (121.28 ± 1.96) mg·g^−1^ with excellent stability, indicating that the extraction process of total flavonoids from *C. retusus* leaf was feasible under the optimal conditions.

**TABLE 2:** Results of orthogonal experiment.

No.	Factors				TFC/mg·g^−1^
	A	B	C	D	
1	1	1	1	1	91.48
2	1	2	2	2	104.35
3	1	3	3	3	107.05
4	2	1	2	3	115.01
5	2	2	3	1	112.58
6	2	3	1	2	114.62
7	3	1	3	2	111.63
8	3	2	1	3	109.83
9	3	3	2	1	109.23
K1	302.88	318.12	315.93	313.29	
K2	342.21	326.76	328.59	330.60	
K3	330.69	330.90	331.26	331.89	
k1	100.96	106.04	105.31	104.43	
k2	114.07	108.92	109.53	110.20	
k3	110.23	110.30	110.42	110.63	
R	13.11	4.26	5.11	6.20	
Optimization scheme	A2	B3	C3	D3	

**TABLE 3:** TFC in *C. retusa* leaf extract obtained under the optimal conditions.

No.	TFC/mg·g^−1^	Average value/mg·g^−1^	RSD%
1	121.02	121.28 ± 1.96	1.61
2	119.47		
3	123.36		

### Purification of total flavonoid

TFC is an important indicator for natural plant extract, which affects the composition and biological activity of the extract. In general, extract containing a higher concentration of flavonoids has better biological activity and economic value. To obtain *C. retusus* leaf extract containing a high content of total flavonoids, the macroporous resin separation method was used to purify total flavonoids. The extract solution was loaded onto a macroporous adsorbing resin column and eluted with 0%, 10%, 20%, 30%, 40%, 50%, and 60% ethanol solution. After freeze-drying, a solid-state extract was obtained. The TFC in the extract was recorded in Supplementary Table S1. The results indicated that the TFC in 0% ethanol solution was too low to be detected. With increasing ethanol concentrations ranging from 10% to 50%, the TFC was increased from 21.01% to 88.51%. It was evident that the content of total flavonoid obtained in the extract from 20%–50% ethanol solution was higher than that in the raw extract. When the eluent was 60% ethanol solution, TFC decreased to 25.74%. These results suggest that most flavonoids had been eluted *via* 20%–50% ethanol solution, and only a few flavonoids were eluted *via* 10% and 60% ethanol solution. The mass and yield of extract after purification is given in Supplementary Table S1. It is clear that most compounds in extract were soluble in water, such as polysaccharide and amino acid. With increased ethanol concentration, the mass of extract after purification decreased, indicating that polar flavone glycoside was greater and weak polar flavonoid aglycones were fewer. As a result, the amount of total flavonoid in the extract was increased after purification by macroporous resin separation. Moreover, extract containing a different content of total flavonoid could be prepared by elution with different concentrations of ethanol solution; the highest content of total flavonoids in extract was up to 88.51%, obtained in 50% ethanol solution.

### DPPH radical scavenging assay

**FIGURE 2:**
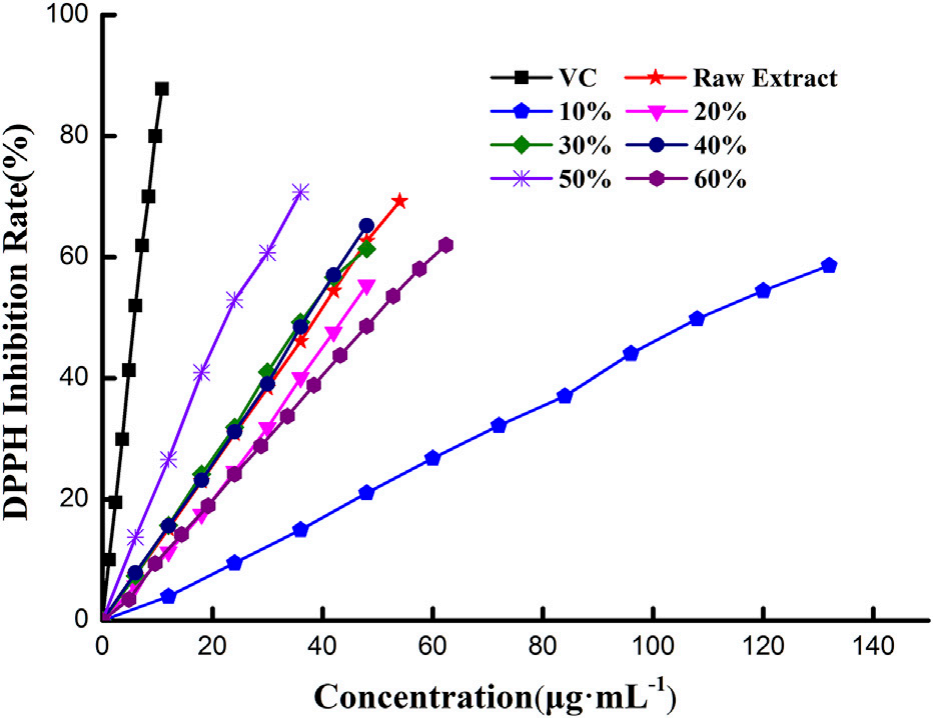
Scavenging activity of *C. retusa* leaf extract on DPPH free radical.

The DPPH radical scavenging activity of extracts purified by macroporous resin separation is assessed in Figure 2 and Table 4. Figure 2 shows that the DPPH inhibition rate went up with increased extract concentration. The curve of extract obtained in 50% ethanol solution rose the fastest in all extracts and was closer to the curve of VC; this indicated that extract obtained in 50% ethanol solution had higher antioxidant capacity. Likewise, extract obtained in 10% ethanol solution had lower antioxidant capacity, while the others had middling capacity. Moreover, the DPPH inhibition rate was proportional to extract concentration, with the calibration curves of the quantitative relationship between the inhibition rate and extract concentration shown in Table 4. Antioxidant activity was expressed as the amount of antioxidant necessary to decrease the initial DPPH concentration for 50% of maximal effect (concentration, EC_50_). EC_50_ of DPPH free radical scavenging activity was calculated from calibration curves (Lu et al., 2019). The EC_50_ of extract obtained in 50% ethanol solution was 24.08 μg·mL^−1^, which was the lowest in all extracts and about four times than that of VC (5.97 μg·mL^−1^). This meant that the antioxidant capacity of extract obtained in 50% ethanol solution was about a quarter of VC. Similarly, the antioxidant capacity of extracts was evaluated by EC_50_; the order of antioxidant capacity was 50% > 40%>30% > raw extract>20% > 60%>10%, which was not the precise order of TFC in extract (50% > 40%>30% > 20%> raw extract >60% > 10%). These results indicate that the antioxidant capacity of extracts was associated with TFC. Moreover, the kind of compounds in extract may also contribute to the antioxidant capacity. Extract obtained in different ethanol solutions had different polarities, which indicated various flavonoids in the extract. When the TFC had a large gap, its effect on antioxidant capacity was significant. When TFC had little difference, the kinds of flavonoids would obviously affect the antioxidant capacity.

**TABLE 4:** Calibration curves and EC_50_ value of *C. retusa* leaf extracts on DPPH free radical.

Samples	Calibration curves	*R* ^2^	EC_50_/μg·mL^−1^
VC	y = 8.2783x + 0.5665	0.9984	5.97
Raw extract	y = 1.2957x − 0.2415	0.9998	38.78
10%	y = 0.4605x − 1.0059	0.9987	110.76
20%	y = 1.1638x − 1.9098	0.9958	44.60
30%	y = 1.3258x + 0.0283	0.9973	37.69
40%	y = 1.361x − 0.6849	0.9991	37.24
50%	y = 1.9803x + 2.3228	0.9916	24.08
60%	y = 1.0155x − 0.4055	0.9995	49.64

## Conclusion

In this study, the extraction process of total flavonoids from *Chionanthus retusus* leaf was optimized by single-factor and orthogonal experiments. The optimal conditions were: ethanol concentration of 60%, ultrasonic power of 140 W, liquid-solid ratio of 25:1 ml g^−1^, and extraction temperature of 80°C. The TFC could reach up to (121.28 ± 1.96) mg·g^−1^ under the optimal conditions. TFC could be increased significantly by macroporous resin separation, with the maximal TFC reaching 88.51%. At the same time, the DPPH radical scavenging activity assay demonstrated that the extracts had a higher antioxidant capacity related to TFC and the kinds of flavonoids. Thus, *C. retusus* leaf extract containing higher total flavonoids could be obtained according to the extraction process and purification method outlined in this study. Extracts containing different TFC were also obtained by the study, which have promise for use in medicines, dietary supplements, and cosmetics.

## Data Availability

The original contributions presented in the study are included in the article/Supplementary Material. Further inquiries can be directed to the corresponding author.
