# Supplementary material for: Extraction and purification of antioxidative flavonoids from Chionanthus retusa leaf
Source: Front Bioeng Biotechnol. 2022 Dec 9;10:1085562. doi: 10.3389/fbioe.2022.1085562 (PMC9780382; doi:10.3389/fbioe.2022.1085562)
Supplement: Supplementary file 1 [file DataSheet1.pdf]

Supplementary Material for

Extraction and Purification of Antioxidantive Flavonoids from *Chionanthus retusa* Leaves

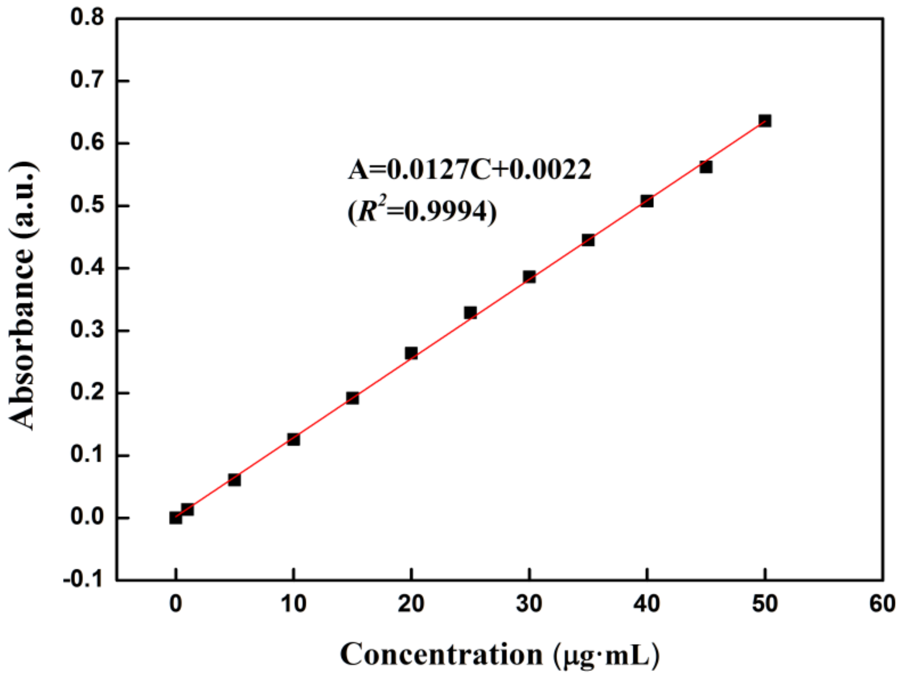

FIGURE S1 | The calibration curve of standard rutin

TABLE S1 | The TFC and mass of *Chionanthus retusa* leaves extract

Ethanol concentration /%	TFC in raw extract /%	Mass of raw extract/g	TFC in extract after purification /%	Mass of extract after purification /g	yield of extract after purification /%
0			—	2.0625	39.32
10			21.01	0.7142	13.62
20			35.46	0.5351	10.20
30	26.56	5.2452	36.30	0.2841	5.42
40			43.62	0.1989	3.79
50			88.51	0.1194	2.28
60			25.74	0.0541	1.03
